# Correction: Superior upconversion fluorescence dopants for highly efficient deep-blue electroluminescent devices

**DOI:** 10.1039/c6sc90033j

**Published:** 2016-05-16

**Authors:** Yi-Hsiang Chen, Chih-Chun Lin, Min-Jie Huang, Kevin Hung, Yi-Ching Wu, Wei-Chieh Lin, Ren-Wu Chen-Cheng, Hao-Wu Lin, Chien-Hong Cheng

**Affiliations:** a Department of Chemistry, National Tsing Hua University Hsinchu 30013 Taiwan chcheng@mx.nthu.edu.tw; b Department of Materials Science and Engineering, National Tsing Hua University Hsinchu 30013 Taiwan

## Abstract

Correction for ‘Superior upconversion fluorescence dopants for highly efficient deep-blue electroluminescent devices’ by Yi-Hsiang Chen *et al.*, *Chem. Sci.*, 2016, DOI: 10.1039/c6sc00100a.

Modified versions of Schemes 1 and 2 are given below in which correct chemical structures for the pyrene groups are shown.

**Scheme 1:**
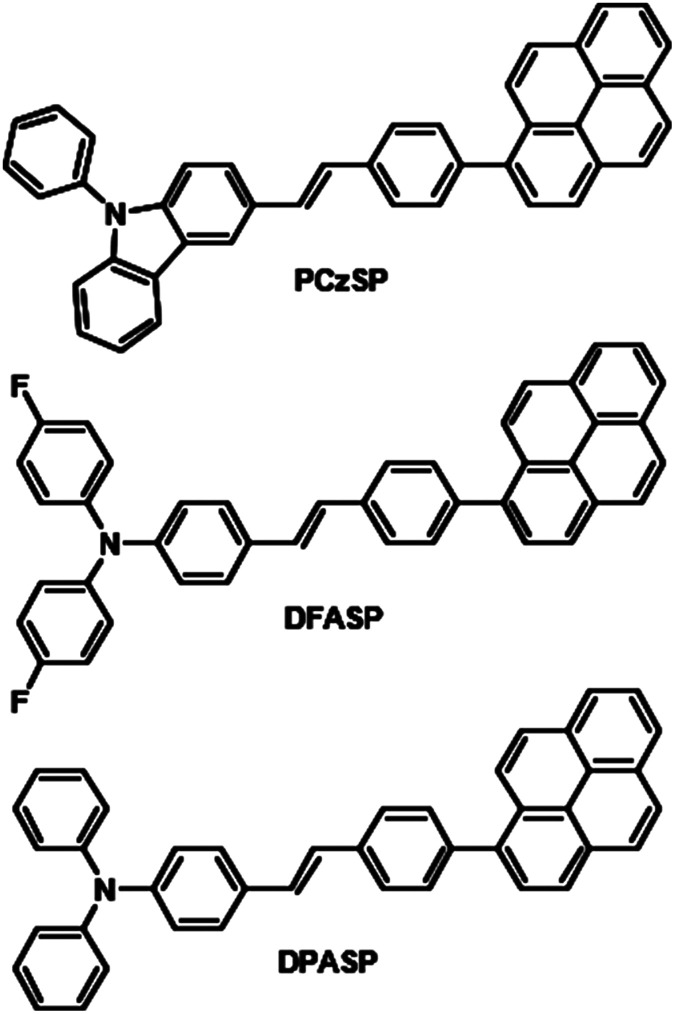
Molecular structures of styrylpyrene-based materials, PCzSP, DFASP and DPASP.

**Scheme 2:**
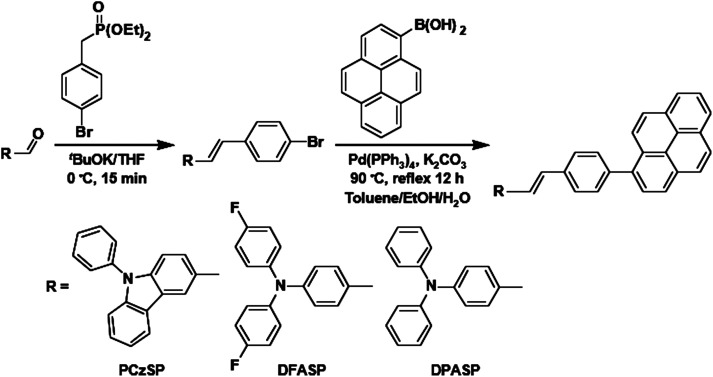
Synthetic routes for PCzSP, DFASP and DPASP.

The Royal Society of Chemistry apologises for these errors and any consequent inconvenience to authors and readers.

